# Limited Antioxidant Effect of Rosemary in Lipid Oxidation of Pan-Fried Salmon

**DOI:** 10.3390/biom9080313

**Published:** 2019-07-28

**Authors:** Kin Sum Leung, Ho Hang Leung, Ching Yu Wu, Jean-Marie Galano, Thierry Durand, Jetty Chung-Yung Lee

**Affiliations:** 1 School of Biological Sciences, The University of Hong Kong, Pokfulam Road, Hong Kong; 2 Institut des Biomolécules Max Mousseron, CNRS, ENSCM Faculté de Pharmacie, Université de Montpellier, 15 Av. Ch. Flahault, BP 14491, F-34093 Montpellier CEDEX 05, France

**Keywords:** salmon, pan-frying, rosemary, lipid oxidation, polyunsaturated fatty acids, aldehydes, isoprostanes

## Abstract

Consumption of omega-3 polyunsaturated fatty acids (n-3 PUFAs) rich fatty fish is known to provide an array of health benefits. However, high temperature in food preparation, such as pan-frying, potentially degrades eicosapentaenoic acid (EPA) and docosahexaenoic acid (DHA) of the n-3 PUFAs by heat oxidation. The addition of antioxidant condiments, and herbs in particular, may retard PUFA peroxidation and preserve EPA and DHA during pan-frying. In this study, different types of antioxidant condiments (sage, rosemary, black peppercorn, thyme, basil, and garlic) were tested for antioxidant capacity, and the condiment with the highest capacity was selected for its effect on lipid oxidation of salmon. The changes in fatty acids and lipid peroxidation of salmon, during pan-frying with the selected condiment (olive oil infused with rosemary, RO_(infused)_), were compared with salmon prepared in extra virgin olive oil, olive oil, or without oil. The total saturated fatty acid was found to be less in pan fried salmon with RO_(infused)_. None of the oil type conserved EPA- and DHA-content in salmon. However, RO_(infused)_ lowered lipid peroxidation by lessening hydroperoxide and 4-HNE formation, but not the other related products (HDHA, HETE, isoprostanes). Our observation indicates that the antioxidant capacity of RO_(infused)_, when it is incorporated with food, becomes limited.

## 1. Introduction

Fatty fish, such as salmon, is rich in long chain omega-3 polyunsaturated fatty acids (n-3 PUFAs), namely eicosapentaenoic acid (EPA) and docosahexaenoic acid (DHA). Numerous researches evidently showed that EPA and DHA are beneficial to the human body, such as in lowering cardiovascular disease, aiding brain development, preventing neurodegenerative diseases, and alleviating the symptoms of rheumatoid arthritis [1,2,3,4,5,6]. EPA and DHA can be converted from plant-based n-3 PUFA e.g., α-linolenic acid (ALA) but the conversion efficacy is low in human metabolism, therefore, it is essential for one to consume sufficient fatty fish for health benefits [7].

Because of food safety reasons, salmon is commonly cooked before consumption. Pan-frying is a popular method of preparing fatty fish, since it is fast, convenient, and can generate typical characteristics of color, flavor, texture, and palatability, which is highly appreciated by consumers [8]. However, pan frying salmon may also cause some undesirable changes [9,10]. The high temperatures generated from pan-frying may breakdown EPA and DHA through lipid oxidation [11,12,13]. Also, studies suggest that the heat from pan-frying, not only increase the oxidation of fatty acids on the surface, but also inside fish meat [8,9,14,15].

It is well-studied that thermal treatment increases the rate of non-enzymatic lipid oxidation in food, and that it generates a mixture of products. For example, F_2_-isoprostanes are formed from arachidonic acid (ARA), which is a known biomarker for oxidative stress [16]. Similarly, F_3_-isoprotanes and F_4_-neuroprostanes are formed from EPA, and DHA, respectively [17,18]. Although increasing numbers of research suggest that F_3_-isoprostanes and F_4_-neuroprostanes are beneficial to human health, due to their cardio-protective and anti-inflammatory properties [19,20], it is not known whether they can be absorbed or provide health benefits when consumed. Furthermore, the production of 4-hydroxy-2-hexenal (4-HHE) from DHA is claimed to be neurotoxic in neuronal cells by augmenting ROS activity and down-regulating the antioxidant enzyme glutathione (GSH) levels [21]. To our understanding the production of these oxidized products will exhaust the salmon EPA and DHA, and consequently affect the nutritional quality of salmon.

Vegetable oils are used to prevent food sticking onto the pan during cooking. However, depending on the type of cooking oil, the fatty acid content is different where monounsaturated fatty acids, such as oleic acid, are predominant in olive oil, whereas n-6 PUFAs are the main fatty acids in corn oil. Aside from heat, the rate of oxidative degradation is greatly affected by the level of fatty acid unsaturation, where the skipped diene structure increases the vulnerability to oxidation [9,22]. Nonetheless, the presence of natural antioxidants in the oil e.g., extra virgin olive oil, may potentially reduce the susceptibilities towards lipid oxidation during pan-frying [22]. It is suggested that herbs and spices are rich in different types of antioxidants and potentially lower lipid oxidation when added to cooking oil. For example, piperine in black pepper was observed to protect against oxidative damage, in several in vivo studies, by inhibiting or quenching free radicals and reactive oxygen species [23,24,25]. Rosemary consists of carnosic acid and carnosol that are shown to inhibit lipid peroxidation in cell culture [26], lower DNA damage [27], and improve antioxidant status in aged rats [28]. Altogether, it is possible that pan-frying salmon with suitable cooking oils and condiments may protect EPA and DHA degradation by lowering the rate of lipid oxidation [29,30].

Although many studies on lipid oxidation in cooked salmon have been reported, the information on cooking salmon with condiments-infused oil is limited. In this study, we (1) investigated the best condiments to infuse into the cooking oil, based on their antioxidant properties, (2) evaluated the changes in fatty acids and lipid oxidation in salmon pan-fried with different cooking oils, and (3) elucidated whether the infusion of condiments in cooking oil preserved EPA and DHA in pan-fried salmon.

## 2. Materials and Methods

### 2.1. Chemicals and Reagents

All organic solvents used were at least analytical grade. Methanol and acetic acid, used in LC-MS/MS analysis, were HPLC grade. Boron trifluoride-methanol solution, used in the sample preparation of gss chromatography-mass spectrometry (GC-MS), was GC grade. The organic solvents and 37-FAME mix were purchased from Sigma Aldrich (St. Louis, MO, USA). Isoprostanoids, hydroxy-docosahexaenoic acid (HDHA), hydroxyeicosatetraenoic aicd (HETE) and resolvin standards were purchased from Cayman Chemical Co. (Ann Arbor, MI, USA) and dihomo-isoprostanes, dihomo-isofurans, neuroprostanes, and neurofurans were synthesized in-house by Institut des Biomolécules Max Mousseron (IBMM, Montpellier, France).

### 2.2. Fish Samples, Cooking Oils, and Condiments

Frozen salmon fillets (with skin) were purchased from a local supermarket and stored at −20 °C before use. Extra virgin olive oil (EVOO) and olive oil (OO) (Bertolli, Italy) were also purchased from a local supermarket. Six different fresh condiments, including sage, rosemary, black peppercorn, thyme, basil, and garlic were selected for the experiment, due to their popularity in common salmon recipes. They were all purchased from a local supermarket, stored in 4 °C, and used for the experiments within 2 days.

### 2.3. Sample Preparation and Analysis

#### 2.3.1. Condiments and Cooking Oils

The condiments were extracted according to a previous study [31] to measure the level of antioxidant activity (Supplementary S1), and the antioxidants in the oils were extracted, according to Ninfali et al. [32] (Supplementary S2). The antioxidant capacity was analyzed by 2,2′-azino-bis(3-ethylbenzothiazoline-6-sulphonic acid) (ABTS) assay, according to Re et al. [33] (Supplementary S3) and the total phenolic content of the condiment extracts was determined by Folin–Ciocalteu assay [34] (Supplementary S4).

#### 2.3.2. Condiment-Infused Oil

**Figure 1:**
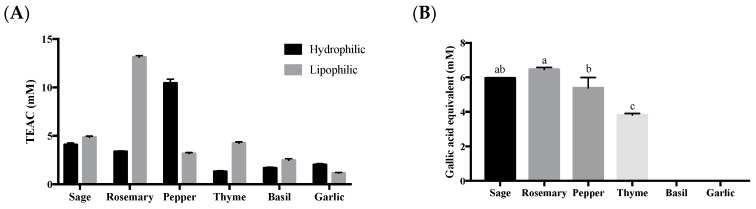
Antioxidant capacity (**A**) and total phenolic content of fresh condiment extract (1:1, hydrophilic: lipophilic) (**B**). Data presented are mean ± S.D. (*n* = 3). Kruskal-Wallis (non-parametric ANOVA) test ranks black pepper and rosemary to be the highest in hydrophilic, and lipophilic extracts, respectively. Columns sharing different alphabets are significantly different at least *p* < 0.05.

The highest lipophilic antioxidant capacity was rosemary, while black pepper showed the greatest hydrophilic antioxidant capacity (Figure 1). Therefore, rosemary and black peppercorn were chosen for condiment-infused oil production. Rosemary was cut into 0.5 cm^2^ and blended into semi-paste by a kitchen blender (BL227, Kenwood, NT, Hong Kong). Black peppercorn was milled by the grinder into powder. Either the rosemary or black pepper was added into olive oil in a ratio of 1:4 (g of condiment to ml of olive oil), heated at low heat (80 ± 5 °C) for 10 min. A mass of 0.5 g oil was collected at 3 time points: Before heating (RO_(initial)_), immediately after heating (RO_(heated)_), and after resting with the rosemary or black peppercorn for 1 hour in room temperature (RO_(infused)_). The collected oils were filtered with 0.45 µm PTFE filter and analyzed for their antioxidant capacity by ABTS assay [33].

#### 2.3.3. Oil from Salmon Meat

**Figure 2:**
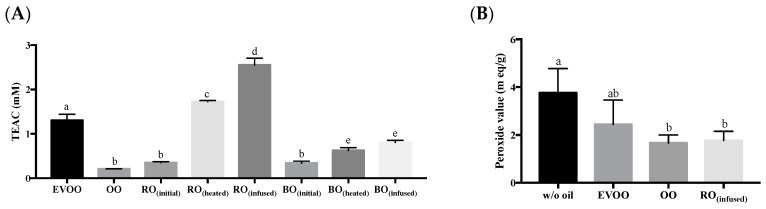
Antioxidant capacity of the cooking oils (**A**) and peroxide value of pan-fried salmon in cooking oils (**B**). Data presented are mean ± S.D. (*n* = 3). EVOO: Extra virgin olive oil; OO: Olive oil; RO_(infused)_: Rosemary-infused oil; BO: Black pepper-infused oil; (initial): Oil collected immediately after addition of condiments; (heated): Oil collected after addition of condiments and heating for 10 min; (infused): Oil collected after addition of condiments, heating for 10 min, and infusion for 1 h. Columns sharing different alphabets are significantly different at least *p* < 0.05.

Before cooking, the salmon meat (50 g each) was thawed at 4 °C overnight and divided randomly into 4 groups: (i) Pan-frying without oil as control; (ii) pan-frying with 5 ml EVOO as a positive control; (iii) pan-frying with 5 mL OO; (iv) pan-frying with 5 mL rosemary-infused oil (RO_(infused)_). Rosemary was chosen as it had the highest antioxidant capacity among the condiments (as shown in Figure 1 and Figure 2A). The fillets were pan-fried on a medium-sized frying pan at 200 °C for 4 min and then flipped for another 4 min cooking. The samples were cooled, finely chopped, and stored at −80 °C until further analysis.

Oil was extracted from a portion of the cooked salmon samples (50 g) with 500 mL of n-hexane/diethyl ether (80:20, vol/vol) for 8 hours using a Soxhlet extractor. The oil collected was cooled to room temperature and dried completely using nitrogen gas. The dried oil was purged with nitrogen and stored at −80 °C until further analysis. The extracted fish oil was used for PV test to measure hydroperoxides, according to Takagi et al. method [35] (Supplementary S5).

#### 2.3.4. Salmon Meat

The antioxidant component of the salmon meat was extracted according to previous study with modifications [36] (Supplementary S2). All extracts were analyzed for the antioxidant capacity by ABTS assay [33]. The fatty acid content in the four groups of treated salmon fillet samples (*n* = 6) were extracted and analyzed by gas chromatography mass spectrometry (GC-MS), according to Quehenberger et al., with modifications [37] (Supplementary S6). Oxidized PUFA products were extracted from the salmon meat samples (*n* = 6) and analyzed by liquid chromatography tandem mass spectrometry (LC-MS/MS), according to Dupuy et al. method [38] (Supplementary S7), while the concentrations of 4-HHE and 4-HNE in salmon meat samples were analyzed by LC-MS/MS, as reported previously with modifications [39] (Supplementary S8).

### 2.4. Statistical Analysis

All data were analyzed by GraphPad Prism (version 6 for Mac, USA) and reported as mean ± S.D. For the differences between groups, significances were analyzed by one-way analysis of variance (ANOVA) and *p*-value <0.05 was considered statistically significant.

## 3. Results and Discussion

Six condiments, that are commonly used for pan-frying salmon, were chosen to test antioxidant capacity [30,40,41,42,43,44,45]. Rosemary showed the highest lipophilic antioxidant capacity (Figure 1A) and phenolic compound levels (Figure 1B), while black pepper had the highest hydrophilic antioxidant capacity and the second highest phenolic compound content. They were, accordingly, selected to be infused in olive oil, considering the hypothetical fact that they may enhance the antioxidant capacity of oil.

Infusing rosemary in OO enriched the antioxidant capacity. The polyphenols, namely lipid soluble carnosic acid of the fresh rosemary, potentially enriched OO for this antioxidant effect [40]. Although our study did not determine carnosic acid in the RO_(infused)_, we observed that rosemary required low temperature heating for adequate infusion of the antioxidant component (as measured by antioxidant capacity) to be absorbed by the olive oil (Figure 2A). On the other hand, as anticipated, black pepper did not increase the antioxidant capacity in olive oil as the potent antioxidant portion was hydrophilic.

Unexpectedly, pan-frying salmon with RO_(infused)_ reduced total fatty acids, when compared to the control group (Table 1). It is plausible the fatty acids from salmon leached into the cooking oil [46] or degraded into volatile compounds. Notwithstanding this, the fatty acid profile of the remaining cooking oil was not measured as a minute amount of cooking oil remained on the pan after frying. Nonetheless, % saturated fatty acids in pan-fried salmon with RO_(infused)_ was significantly lowered than those cooked without oil, while the % n-6 PUFAs in pan-fried salmon with EVOO significantly decreased. Moreover, both OO and EVOO are rich in oleic acid (C18:1n9) but the levels were not elevated in salmon pan-fried with OO, EVOO or RO_(infused)_. It was observed by Sioen et al. [46], OO formed a crust on the fish during cooking and, thus, prevents the salmon meat form absorbing exogenous oil that could affect the fatty acid composition. In line with the findings in this study, the group also reported pan-frying salmon with OO did not significantly alter the fatty acid profile.

During pan-frying, the high temperature accelerates the lipid peroxidation process and generates an array of mixed lipid oxidized products. In this study, both primary, intermediate, and secondary oxidation products were evaluated. A remarkable suppression in primary lipid peroxidation was observed in pan-fried salmons, prepared in OO and RO_(infused)_, where the peroxide values were significantly lower, compared to the control group (Figure 2B). Since the peroxide values of salmon pan-fried with OO and RO_(infused)_ were similar, the reduction in primary lipid peroxidation did not appear to be attributed to the antioxidant component.

**Figure 3:**
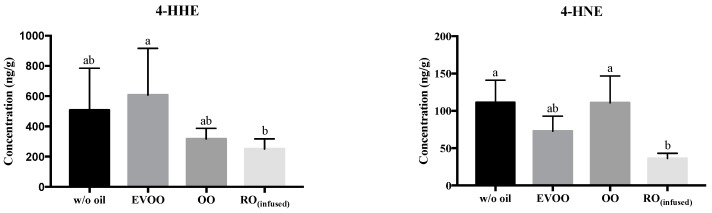
Level of 4-HHE and 4-HNE in pan-fried salmon (ng of analytes per g of salmon meat). Data presented are mean ± S.D. (*n* = 6). w/o oil: without oil; EVOO: extra virgin olive oil; OO: olive oil; RO_(infused)_: rosemary-infused oil. Columns sharing different alphabets are significantly different at least *p* < 0.05.

Hydroperoxides are a mix of primary and intermediate lipid peroxidation products that are unstable and decompose into a wide range of volatile flavor compounds and non-volatile products, or further oxidize into secondary oxidation products, such as HNE and HHE [47]. In correspondence, RO_(infused)_ reduced the formation of aldehydes, especially 4-HNE during pan-frying of salmon (Figure 3). 4-HHE and 4-HNE are some of the end products of n-3, and n-6 PUFAs oxidation, respectively. Both are considered toxic lipid peroxidation products, however, in low concentrations, they showed cardioprotective effects. Low concentrations of 4-HHE protected endothelial cells through antioxidant activation of Nrf2 mediated HO-1 expression [48], while low concentrations of 4-HNE prevented cardiac ischemia-reperfusion injury by activating Nrf2 and the subsequent stimulation of glutathione biosynthesis [49]. Nevertheless, these aldehydes in excess, promote cross-linking with protein, form adducts in vivo, cause mitochondrial DNA mutation, as well as mitochondrial dysfunction [50,51]. Moreover, augmented levels of these adducts were found in human atherosclerotic lesions and in the spinal cords of patients with amyotrophic lateral sclerosis [50], and HNE has been associated to neurological diseases, cancer, diabetes, and CVD [51].

**Figure 4:**
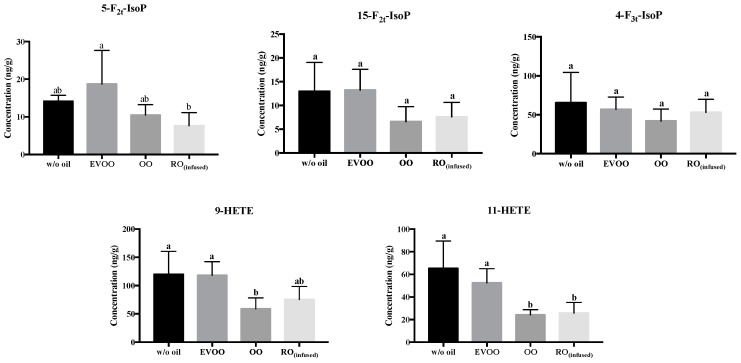
Concentration of non-enzymatic oxidized products of n-6 polyunsaturated fatty acid (PUFA) in pan-fried salmon samples (ng of analytes per g of salmon meat). F_2_-isoprostanes and HETEs are derived from arachidonic acid (ARA) and 4-F_3t_-IsoP is derived from n-6 DPA. Data presented are mean ± S.D. (*n* = 6). w/o oil: without oil; EVOO: extra virgin olive oil; OO: olive oil; RO_(infused)_: rosemary-infused oil. Columns sharing different alphabets are significantly different at least *p* < 0.05.

**Figure 5:**
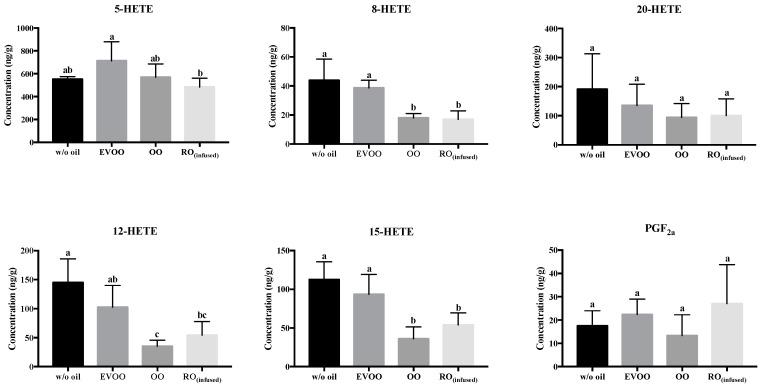
Concentration of enzymatic oxidized products of ARA in pan-fried salmon samples (ng of analytes per g of salmon meat). Data are presented in mean ± S.D. (*n* = 6). w/o oil: without oil; EVOO: extra virgin olive oil; OO: olive oil; RO_(infused)_: rosemary-infused oil. Columns sharing different alphabets are significantly different at least *p* < 0.05.

As indicated, carnosic acid in rosemary is a potent lipophilic antioxidant. In an in vitro study using carnosic acid, Masuda et al. [52] explicitly reported that the two reactive phenolic groups in the aromatic ring of the structure were responsible for the antioxidant activity. When salmon was pan-fried with OO, two non-enzymatic n-3 PUFAs intermediate oxidized products; 8-F_3t_-Isoprostane derived from EPA, and 8-HDHA derived from DHA, were significantly reduced (Supplementary Figure S1). Pan-frying salmon with OO also showed a suppressing effect on two non-enzymatic n-6 PUFAs intermediate oxidized products; 9-HETE and 11-HETE from ARA (Figure 4). However, this does not imply that the formation of free radicals or reactive oxygen species (ROS) are inhibited because the oxidative stress biomarker, i.e., the level of F_2t_-Isoprostane (although low in concentration) [14], was not substantially reduced. Nonetheless, RO_(infused)_ did not protect against lipid peroxidation in pan-fried salmon, and the lipid peroxidation products were at similar levels as the control. During the frying process, temperature-sensitive lipoxygenase enzyme was expected to be inactivated by the high temperatures [21]. Despite this presumption, it was surprising that the lipoxygenase-mediated intermediate oxidized lipid products, including 8-, 12- and 15-HETE in pan-fried salmon, while OO and RO_(infused)_ were reduced (Figure 5). It is possible that the formation of HETE racemics may have reduced non-enzymatic metabolite generation, and/or 12- and 15-HETE were further oxidized into down-stream metabolites that are not cytotoxic [53].

It was anticipated that the effect of RO_(infused)_ in reducing lipid peroxidation would be more distinguishable. In previous studies, it showed excellent antioxidant properties and prevented lipid peroxidation. Some studies reported that rosemary delays n-3 PUFA peroxidation of salmon in frozen storage [41,54]. However, the addition of carnosic acid to virgin olive oil did not inhibit lipid oxidation, and in fact augmented both primary and secondary oxidation products when used for deep-frying food [55]. In this study, the addition of RO_(infused)_ did not effectively inhibit lipid oxidation in salmon, yet, primary, intermediate, and secondary oxidation products did not elevate either. The difference in cooking temperature and time resulted in contrasting findings with other research. Further, it is likely that carnosic acid underwent thermal degradation during the cooking process, thereby reducing the protection mechanism of lipid peroxidation by RO_(infused)_.

In conclusion, RO_(infused)_ reduced the saturated fatty acids of the salmon meat. It did not alter the intermediate oxidized products (F_3_-isoprostanes, F_4_-neuroprostanes, HDHA) in pan-fried salmon that are health benefiting, instead, it lowered the development of toxic aldehydes of ARA and DHA.

## Figures and Tables

**Table 1:** Fatty acids content in pan-fried salmon (µg of analytes per g of salmon meat) cooked with or without extra virgin olive oil (EVOO), olive oil (OO), or rosemary-infused oil (RO_(infused))_.

	Salmon (*n* = 6)			
	w/o oil	EVOO	OO	RO_(infused)_
Total	14034.2 ± 1856.9 ^a^	14651.7 ± 3588.7 ^a^	12140.9 ± 1059.4 ^a^	9869.2 ± 1714.8 ^b^
∑ SFA (%)	8.69 ± 0.14 ^a^	8.76 ± 0.18 ^a^	8.45 ± 0.30 ^ab^	7.94 ± 0.73 ^b^
∑ MUFA (%)	25.50 ± 1.14 ^a^	24.91 ± 1.09 ^a^	24.75 ± 2.01 ^a^	23.38 ± 1.10 ^a^
∑ n-6 PUFA (%)	20.74 ± 0.65 ^a^	19.11 ± 0.95 ^b^	20.00 ± 0.52 ^ab^	19.95 ± 1.33 ^ab^
∑ n-3 PUFA (%)	45.06 ± 1.67 ^a^	47.22 ± 1.82 ^a^	46.81 ± 2.51 ^a^	48.72 ± 3.13 ^a^

Data presented are mean ± S.D. w/o oil: Salmon pan-fried without oil; EVOO: Salmon pan-fried with EVOO; OO: Salmon pan-fried with olive oil; RO_(infused)_: Salmon pan-fried with rosemary-infused oil. Values sharing different alphabets are significantly different at least *p* < 0.05.

## Supplementary Materials

The following are available online at https://www.mdpi.com/2218-273X/9/8/313/s1.
