# Supplementary material for: Limited Antioxidant Effect of Rosemary in Lipid Oxidation of Pan-Fried Salmon
Source: Biomolecules. 2019 Jul 28;9(8):313. doi: 10.3390/biom9080313 (PMC6723415; doi:10.3390/biom9080313)
Supplement: Supplementary file 1 [file biomolecules-09-00313-s001.pdf]

Supplementary materials:

Limited antioxidant effect of rosemary in lipid oxidation of pan-fried salmon

Kin Sum Leung[1], Ho Hang Leung[1], Ching Yu Wu[1], Jean-Marie Galano[2], Thierry Durand[2], Jetty Chung-Yung Lee[1*]

[1] School of Biological Sciences, The University of Hong Kong, Pokfulam Road, Hong Kong SAR;
[2] Institut des Biomolécules Max Mousseron, CNRS, ENSCM Faculté de Pharmacie, Université de Montpellier, 15 Av. Ch. Flahault BP 14491 F-34093 Montpellier Cedex 05 France;
* Correspondence: jettylee@hku.hk; Tel.: +852- 2299 0318

S1. Extraction of antioxidants from condiments

The condiments were extracted according to previous study [1] for measurement of antioxidant activity. Briefly, 0.1 g of grounded condiments were extracted with 4 mL of Milli-Q water under agitation for 15 min at room temperature, centrifuged at 1000 × g for 10 min and the supernatant was collected. The extraction was repeated with 2 mL of Milli-Q water and the supernatants were combined. To the residue, acetone was added, and the extraction procedure was repeated to extract lipophilic antioxidants. The water (hydrophilic) extract and acetone (lipophilic) extract were analyzed for antioxidant activity and total phenolic content.

S2. Extraction of antioxidants from cooking oils and salmon meat

The antioxidants of the oils were extracted according to previous study [2]. A mass of 0.5 g oil was diluted (1:1, wt/vol) with 80% methanol, then vortexed for 2 min at room temperature. After centrifugation for 10 min at 5000 × g, the supernatant was collected. The extraction was repeated to ensure all the antioxidants from the cooking oil were extracted. The supernatants were combined and diluted with 0.075 M Na-K phosphate buffer (1:20, vol/vol, pH 7.0) then, filtered with 0.22 μm Millipore filter and analyzed for the antioxidant capacity.

Antioxidant component of the salmon meat was extracted according to previous study with modifications [3]. In brief, 0.1 g of finely chopped salmon sample was homogenized with Folch solution (chloroform/methanol 2:1 vol/vol + 0.05% butylated hydroxytoluene (BHT)) at a ratio of 1:10 (wt/vol). The homogenate was then centrifuged at 2000 × g for 20 min. All extracts were analyzed for the antioxidant capacity

S3. Measurement of trolox equivalent antioxidant capacity (TEAC)

The 2,2'-azino-bis (3-ethylbenzothiazoline-6-sulphonic acid) (ABTS) assay was performed according to Re et al. [4]. In brief, ABTS radical cation (ABTS•+) solution was prepared by reacting 7mM ABTS solution with 2.45 mM potassium persulfate. The mixture was kept in dark at room temperature for 12-16 hours before use. ABTS working solution was obtained by diluting the ABTS radical cation (ABTS•+) solution with 90% methanol to a spectrophotometric (Hitachi, Tokyo Japan) absorbance of 0.70 (±0.02) at 734 nm. Trolox (2.5 mM) was prepared with methanol and used as the standard. A volume of 10 μL sample solution or Trolox standard was added to 1mL ABTS•+ solution for the analysis. Absorbance was taken after 5min. All determinants were carried out in triplicates. Results are expressed as TEAC mM per g for all the samples.

S4. Measurement of total phenolic content (TPC)

The total phenolic content of the condiment extracts was determined by Folin–Ciocalteu assay [5]. Gallic acid was used as the standard. A volume of 100 μL condiment extract (1:1, hydrophilic:lipophilic) was added to 200 μL Folin–Ciocalteu reagent (1:10, reagents: Milli-Q water) and vortex thoroughly. A volume of 800 μL of sodium carbonate (700 mM) was then added to the sample mixture or gallic acid to prevent air oxidation of the phenolic compounds.

The mixture was incubated in dark at room temperature for 1.5 hours. A volume of 200 μL of the mixture was transferred to 96-well microplate and the absorbance was taken at 765 nm using iMark microplate reader (Bio-Rad, Hercules, CA USA).

S5. Measurement of peroxide value (PV)

The extracted fish oil was used for PV test to measure hydroperoxides which are the primary lipid peroxidation products. The PV was measured according to Takagi et al. method [6]. Briefly, 1.5 g of the oil sample was weighed in an Erlenmeyer flask (125 mL) and dissolved in 30 mL acetic acid/ chloroform (3:2 vol/vol). A volume of 0.5 mL saturated potassium iodide solution was added to the dissolved oil, swirled for 1 min and then diluted with 30 mL Milli-Q water. Starch indicator (1 mL) was added to the mixture and titrated with 0.01 M sodium thiosulphate until the dark blue color disappeared.

S6. Measurement of total fatty acid content by gas chromatography mass spectrometry (GC-MS)

The fatty acid content in 4 groups of salmon fillet samples (n=6) were analyzed according to Quehenberger et al. with modifications [7]. In brief, 0.05 g finely chopped salmon was homogenized in 900 μL methanol with internal standard (0.25 μg/μL C19:0 nonadecanoic acid) and then sonicated for 1min. Methanol (1 mL) and 50 μL 1N HCl were then added to the mixture to acidify the sample. Alkaline hydrolysis was performed by adding 500 μL 1N KOH and the mixture was incubated at room temperature for 1 hour. Liquid-liquid extraction was performed by adding 1 mL iso-octane. The mixture was vortexed and centrifuged at 2000 × g for 5 min to induce phase separation. The procedure was repeated to increase the yield of the extraction. The combined extracts were dried at 37°C under a stream of nitrogen. Chemical derivatization was performed by adding 1mL boron trifluoride (BF_3) to the extract, which was then incubated at 60°C for 30 min. To collect the methylated fatty acids, 2 mL hexane: dimethylether (80/20, vol/vol) was added to the mixture, which was vortexed and centrifuged at 2000 × g for 5 min to initiate phase separation. The extraction was repeated to increase the yield. The combined extracts were dried at 37°C under a stream of nitrogen. The dried extracts were re-suspended with 50 μL dichloromethane, and immediately analyzed by GC-MS.

The derived samples were analyzed by an Agilent 5977A mass selective detector coupled to an Agilent 7890B gas chromatograph (Agilent, Santa Clara, CA USA) equipped with an autosampler and computer workstation. The injection port and GC-MS interface were maintained at 240°C and 250°C, respectively. The mass spectrometer was set at negative ionization (NEI) mode. Separation was performed on a SP®-2560 capillary column (100 m x 0.25 mm, df 0.20 μm, Sigma-Aldrich, St. Louis, MO USA). Helium was used as the carrier gas with a flow rate of 1 ml/min. The derivatized sample (1 μl) was injected into the GC inlet at a split ratio of 1:100. The column temperature was maintained at 140°C for 5 min, then raised to 180°C at 8°C per min, and further raised to 210°C at 4°C per min and 250°C at 20°C per min, and then maintained for 10 min. Selective ion monitoring (SIM) and full scan modes were performed to monitor fatty acid methyl ester ions. Quantification was achieved by correlating the peak area of a single fatty acid methyl ester with the C19:0 internal standard peak area. A calibration curve was set using a 37-component FAME mixture in a concentration range of 2 to 400 μg/m. Each curve point was determined in triplicate and the regression coefficient of the calibration curves were between 0.988 and 0.997.

S7. Measurement of PUFA oxidation products by liquid chromatography tandem mass spectrometry (LC-MS/MS)

Oxidized PUFA products were extracted from the salmon meat samples (n=6) prepared in Section 2.3.4 according to Dupuy et al. method [8]. Briefly, 0.1 g finely chopped salmon was homogenized in 10 mL of Folch solution (chloroform/methanol, 2:1 vol/vol + 0.05% BHT). The extraction was performed with orbital shaker on ice for 1 hour. Afterwards, 2 mL 0.9% NaCl

was added to introduce phase separation. The mixture was then shaken on ice for 30 min and centrifuged at 2000 × g for 10 min in room temperature. The lower chloroform phase was collected in a 30 mL glass bottle. Overnight alkaline hydrolysis at room temperature was performed by adding potassium hydroxide (1 M in methanol) and internal standard mix (0.1 ng/μL in methanol) to the extracted sample and then terminated by the addition of 1 M hydrochloric acid. The oxidized PUFA products were purified using mixed anion solid phase extraction (MAX SPE, Waters, Milford, MA USA). The SPE column was preconditioned with 100% methanol and 20 mM formic acid. After loading the sample, it was washed with 2% ammonium hydroxide and 20 mM formic acid. The final eluent was collected with hexane/ethanol/acetic acid (70/29.4/0.6, vol/vol/vol). The samples were then dried at 37°C under a stream of nitrogen gas until complete dryness. It was re-suspended in 100 μL methanol and filtered through 0.45 μm PTFE filter to remove insoluble impurities, and then immediately analyzed by LC-MS/MS.

The Sciex X500R QTOF system (Sciex Applied Biosystems, MA, USA) consisted of an Exion LC liquid chromatograph with a C18 column (150x2.1 mm, 2.6 μm particle size, Phenomenex, Torrance, CA USA) was maintained at 40°C for analysis. The flow rate was set to 300 μl/min and the injection volume was 10 μl. A mobile phase consisting of 0.1% aqueous acetic acid (A) and 0.1% acetic acid/methanol (B) was used. The gradient conditions were maintained at 20% of solvent B for 2 min and between 20% and 98% for 8 min, and then held for 5 min. The percentage of solvent B was reduced to 20% in 1 min and held for an additional 5 min to equilibrate to the initial conditions. The X500R QTOF system was operated in negative electrospray ionization (ESI) mode. The spray voltage was set to -4500V and nitrogen was the curtain gas. The ionization chamber temperature was set at 350°C, and the pressure of the ion source gases 1 and 2 were 35 and 45 psi, respectively. The declustering potential (DP) was set to -80V and the collision energy (CE) was -10V for the QTOF MS. The scan mode was set to multiple reaction monitoring (MRM). All data collected by the X500R QTOF system was analyzed by the Sciex operating system (version 1.2.0.4122). The quantification of each analyte was determined by correlating the peak area to its corresponding deuterated internal standard peak. For analytes without corresponding deuterated internal standards, the following deuterated internal standards, 5(*S*)-HETE-d_8, 15-F_{2t}-IsoP-d_4 and 4-(*RS*)-4-F_{4t}-NeuroP-d_4 were used for quantification. In this study, a total of 34 oxidized PUFA products were identified.

S8. Measurement of 4-hydroxy-2(E)hexenal (4-HHE) and 4-hydroxy-2-nonenal (4-HNE) by liquid chromatography tandem mass spectrometry (LC-MS/MS)

Concentrations of 4-HHE and 4-HNE in salmon fillet samples were analyzed as reported previously with modifications [9]. In brief, 0.1 g of salmon meat samples (n=6 per group) were homogenized with 200 μL BHT solution (1 mg/mL in ethanol) and 2.2 mL ethanol water (50:50, vol/vol). A volume of 100 μL 4-HHE-d_4 with 4-HNE-d_4 (0.1 ng/μL in methanol) internal standards was then added and vortexed with the homogenates. Afterwards, the samples were centrifuged at 2100 × g for 10 min. A volume of 2 mL supernatant was taken and added to 2 mL 0.05 M DNPH solution in acetonitrile/acetic acid (9:1, vol/vol). The derivatization was initiated by incubating the samples in 60°C water bath for 2 hours. To collect the derivatized aldehydes, 4 mL milli-Q water and 2mL hexane were added to the mixture. The mixture was vortex and centrifuged at 2000 × g for 2 min to achieve phase separation. Extraction was repeated with 2 mL hexane to increase the yield. The extracts were combined and dried at 37°C under a stream of nitrogen. The dried extracts were re-suspended with 100 μL 0.1% acetic acid/acetonitrile (60:40, v/v), and analyzed by LC-MS/MS.

Quantification of the derivatized 4-HHE and 4-HNE was performed using Exion LC liquid chromatograph and Sciex X500R QTOF system. The HPLC system was coupled to a Kinetex C18 column (150 x 2.1 mm, 2.6 um, Phenomenex, Torrance, CA USA) and heated to 40 °C for stable separation. The mobile phase was 0.1% acetic acid in water (A) and 0.1% acetic

acid in methanol (B) at a flow rate of 0.3 mL/min. The gradient elution was as follows: 40% to 65% B for the first 8.5 min, then gradually increased to 100% B for 4 min and held for 7.5 min, then decreased to 40% B within 2 min and maintained for 6 min. Analysis was performed in MRM high resolution mode with negative electrospray ionization at a source temperature of 500 °C. The quantification of each analyte was determined by correlating the peak area to its corresponding deuterated internal standard peak.

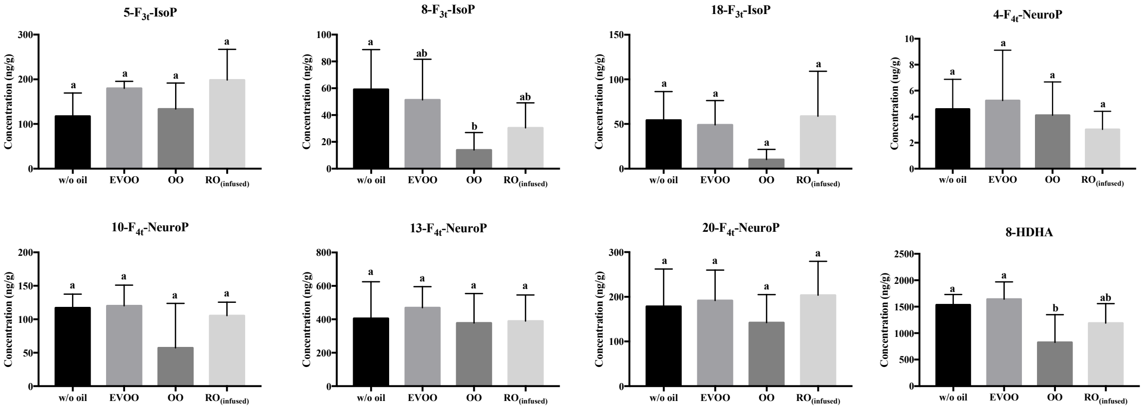

Figure S1. Concentration of non-enzymatic oxidized products of n-3 PUFA in pan-fried salmon samples (ng of analytes per g of salmon meat). All F_3-isoprostanes are derived from EPA while all F_4-neuroprostanes and HDHA are derived from DHA. Data are presented in mean ± S.D. (n=6). w/o oil: without oil; EVOO: extra virgin olive oil; OO: olive oil; $RO_{(infused)}$: rosemary-infused oil. Columns sharing different alphabets are significantly different at least $p < 0.05$.

References

1. Pellegrini, N.; Serafini, M.; Colombi, B.; Del Rio, D.; Salvatore, S.; Bianchi, M.; Brighenti, F. Total antioxidant capacity of plant foods, beverages and oils consumed in Italy assessed by three different in vitro assays. *J Nutr* **2003**, *133*, 2812-2819, doi:10.1093/jn/133.9.2812.
2. Ninfali, P.; Aluigi, G.; Bacchiocca, M.; Magnani, M. Antioxidant capacity of extra-virgin olive oils. *Journal of the American Oil Chemists' Society* **2001**, *78*, 243-247, doi:10.1007/s11746-001-0252-9.
3. Ochrem A., Z.P., Żychlińska-Buczek J., Maj D., Czerniejewska-Surma B., Pokorska J., Kułaj D. Antioxidant capacity and lipid peroxidation products of carp (Cyprinus carpio L.) meat stored in refrigeration conditions with addition of herbs or vegetables. *Czech J. Food Sci.* **2017**, *35*, 251-258.
4. Re, R.; Pellegrini, N.; Proteggente, A.; Pannala, A.; Yang, M.; Rice-Evans, C. Antioxidant activity applying an improved ABTS radical cation decolorization assay. *Free Radic Biol Med* **1999**, *26*, 1231-1237.
5. Ainsworth, E.A.; Gillespie, K.M. Estimation of total phenolic content and other oxidation substrates in plant tissues using Folin-Ciocalteu reagent. *Nat Protoc* **2007**, *2*, 875-877, doi:10.1038/nprot.2007.102.
6. Takagi, T.; Mitsuno, Y.; Masumura, M. Determination of peroxide value by the colorimetric iodine method with protection of iodide as cadmium complex. *Lipids* **1978**, *13*, 147-151, doi:10.1007/BF02533257.
7. Quehenberger, O.; Armando, A.M.; Dennis, E.A. High sensitivity quantitative lipidomics analysis of fatty acids in biological samples by gas chromatography-mass spectrometry. *Biochim Biophys Acta* **2011**, *1811*, 648-656, doi:10.1016/j.bbalip.2011.07.006.
8. Dupuy, A.; Le Faouder, P.; Vigor, C.; Oger, C.; Galano, J.M.; Dray, C.; Lee, J.C.; Valet, P.; Gladine, C.; Durand, T., et al. Simultaneous quantitative profiling of 20 isoprostanoids from omega-3 and omega-6 polyunsaturated fatty acids by LC-MS/MS in various biological samples. *Anal Chim Acta* **2016**, *921*, 46-58, doi:10.1016/j.aca.2016.03.024.
9. Douny, C.; Bayram, P.; Brose, F.; Degand, G.; Scippo, M.L. Development of an LC-MS/MS analytical method for the simultaneous measurement of aldehydes from polyunsaturated fatty acids degradation in animal feed. *Drug Test Anal* **2016**, *8*, 458-464, doi:10.1002/dta.2013.
